# A212 LIVER TRANSPLANTATION FOR PRIMARY SCLEROSING CHOLANGITIS (PSC): EXPERIENCE FROM THE LAST THREE DECADES

**DOI:** 10.1093/jcag/gwab049.211

**Published:** 2022-02-21

**Authors:** J Zhu, D R Lim, L J James, E M Yoshida, K M Peltekian

**Affiliations:** 1 Dalhousie University, Halifax, NS, Canada; 2 Division of Gastroenterology, University of British Columbia, Vancouver, BC, Canada; 3 ATLANTIC HEPATOLOGY SERVICES INC, Halifax, NS, Canada; 4 Gastroenterology, Dalhousie University, Halifax, NS, Canada

## Abstract

**Background:**

In liver transplantation (LT) for PSC, pre-transplant colectomy may lower the risk of PSC recurrence (rPSC), and well-controlled or lack of IBD may protect against rPSC, however, these findings are not consistent across all studies. It is unknown whether recipient/donor sex plays a role in rPSC.

**Aims:**

To study factors associated with PSC recurrence in the post-transplant population.

**Methods:**

This is a retrospective study on adults who received an LT for PSC in Halifax NS from 1985 to 2020. Graft and patient outcomes are analyzed by logistic regression models.

**Results:**

A total of 92 patients underwent deceased donor LT for PSC with a mean follow-up time of 9.2 yrs (SD 7.1). Fifty-three remain active in the program, 2 were lost to follow up and 37 died. The mean age at transplant was 43 (SD13, range 18.9–67.8). Seven patients had a second transplant. The five- and 10-year patient survival rate of the entire cohort was 78.7% and 66% respectively. In the active cohort, the prevalence of rPSC was 23.5% (12/51). Retransplantation for rPSC occurred in 8.3% (1/12). The prevalence of IBD was 82.4% (42/51), consisted of ulcerative pancolitis (64.7%, 33/51), ulcerative proctitis (3.9%), left-sided UC (2%) and Crohn’s disease (9.8%). Males are much more likely than females to undergo a colectomy at any time (OR 5.5, 95% CI 1.07–28.22, p 0.041). Refractory IBD was the predominant indication for a colectomy (10/16), followed by dysplasia or colon cancer (6/16). In the post-transplant period, 69% had stable IBD without therapy escalation, 11.9% were escalated to a biologic, 19% underwent a colectomy for active IBD symptoms. Pre-transplant colectomy negatively predicted rPSC, in this subgroup 0% (0/6) developed rPSC. Neither recipient sex (OR 1.14, 95% CI 0.26–5.0, p 0.86) or recipient age predicted the likelihood of rPSC. There was no association between donor sex on rPSC (OR 1.25, 95% CI 0.30–5.27, p 0.76). A trend towards increased rPSC was observed in male donors to female recipients versus female-to-female transplants (OR 6, 95% CI 0.33–107.42, p 0.224). Overall, having a post-transplant colectomy, subtotal or total, irrespective of timing, did not significantly impact rPSC (OR 0.389, 95% CI 0.09–1.66, p 0.20). Diagnosis of IBD was not associated with an increased risk of rPSC (OR 1.21, p 0.83).

**Conclusions:**

Several factors were associated with rPSC after liver transplant in patients with PSC and IBD, pre-transplant colectomy was found to be protective, male donor to female recipient was a potential risk factor. It is important to study these factors in multi-centered cohorts to understand the pathogenesis of PSC. Pre-transplant total colectomy may be beneficial for several reasons, reducing rPSC, controlling IBD activities, and lowering dysplasia and colon cancer rates in the post-transplant population.

**Funding Agencies:**

None

